# Influence of the Degree of Hydrolysis on Functional Properties and Antioxidant Activity of Enzymatic Soybean Protein Hydrolysates

**DOI:** 10.3390/molecules27186110

**Published:** 2022-09-19

**Authors:** Monirul Islam, Yatao Huang, Serajul Islam, Bei Fan, Litao Tong, Fengzhong Wang

**Affiliations:** 1 Institute of Food Science and Technology, Chinese Academy of Agricultural Sciences, No. 2 Yuan Ming Yuan West Road, Beijing 100193, China; 2 Rural Development Academy (RDA), Bogura 5842, Bangladesh; 3 Key Laboratory of Agro-Products Processing, Ministry of Agriculture and Rural Affairs, Chinese Academy of Agricultural Sciences, Beijing 100193, China; 4 State Key Laboratory of Food Science and Technology, Jiangnan University, Wuxi 214122, China

**Keywords:** soybean protein hydrolysate, enzymatic hydrolysis, degree of hydrolysis, functional properties, antioxidant activity

## Abstract

Soybean protein hydrolysates were prepared using two proteolytic enzymes (Alcalase and Protamex) and the degree of hydrolysis (DH) and their functional and antioxidant properties were evaluated. The highest DH value was 20%, with a yield of 19.77% and protein content of 51.64%. The total amino acid content was more than 41% for all protein hydrolysates. The protein hydrolysates from Protamex at pH 2.0 had excellent solubility, emulsifying activity, and foaming capacity, at 83.83%, 95.03 m^2^/g, and 93.84%, respectively. The water-holding capacity was 4.52 g/g for Alcalase, and the oil-holding capacity was 4.91 g/g for Protamex. The antioxidant activity (62.07%), as measured by the samples’ reaction with DPPH (2,2-diphenyl-1-picrylhydrazyl) and the reducing power (0.27) were the strongest for Protamex. An ABTS activity rate of 70.21% was recorded for Alcalase. These findings indicated a strong potential for the utilization of soybean protein hydrolysates to improve the functional properties and antioxidant activity of soybeans as well as their nutritional values.

## 1. Introduction

Soybean (*Glycine max* L.) is an herbaceous legume plant in the pea family, Leguminosae, subfamily, Papilionnidea, mainly native to East Asia, China, and Japan. It offers desirable functional properties at a low cost and provides a complete protein with high nutritional value, as well as complex carbohydrates, polyunsaturated fatty acids, soluble fiber, and isoflavones [1,2]. Wang et al. [3] described 60 varieties of soybeans that are used for soy products, and 398 million tons of soybeans were produced worldwide in 2018 [2]. Fiala [4] predicted that 72% more protein will be needed by 2030 because of the increasing population, meat consumption, urbanization, and industrialization. 

Protein hydrolysates have been prepared from various sources, including soybean, mung bean, wheat, shrimp, fish, sesame, and some food wastes [5,6,7]. The methods involved in hydrolyzing food proteins have included physical, chemical, and biological processes [8,9,10]. Enzymatic modification is a safe and effective method to improve the functional properties of soybean protein, as it simplifies the operational conditions, reduces the creation of by-products, and is more environmentally friendly [11]. Protein hydrolysates and peptides can be prepared via hydrolysis with proteolytic enzymes. Amino acid profiles, functional, and antioxidant properties are highly influenced by hydrolysis conditions, including digestion time, pH, temperature, buffer-to-substrate ratio, and enzyme-to-substrate ratio (E/S). Proteolytic enzymes such as Alcalase, Papain, Protamex, Flavourzyme, Neutrase, Bromelain, Pancreatin, Trypsin, Chymotrypsin, and Thermolysin are the most frequently used commercial applications for enzymatic hydrolysis. Proteolytic enzymes cleave a protein into peptide fragments, composed of 2–20 amino acids [12]. 

Functional properties of protein hydrolysates such as solubility, emulsification, and foaming, among others, are important to the food industry, as the functional quality and availability affect their application as ingredients in specific foods [13]. The enzymatic hydrolysis of proteins is a powerful tool in the modification of their functional properties for use in specific food applications [14]. Islam, Wang, Admassu, Sulieman, and Wei [12] reported that protein solubility was the most important functional property, as it influenced the emulsifying and water-binding capacity and the foaming property. Minh [15] found that the Protamex digestion of soy protein produced the branched-chain amino acids leucine (1.15%), isoleucine (0.31%), and valine (0.34%), while digestion with the Alcalase enzyme resulted in leucine (0.96%), isoleucine (0.44%), and valine (0.46%) from soybean protein hydrolysates. The hydrolyzed soybean protein content using the Protamex enzyme was 22.9%. 

The enzymatic hydrolysis of proteins can improve solubility as well as other physicochemical properties and can lead to the generation of bioactive peptides [16]. Many researchers and manufacturers tend to prefer using enzymatic hydrolysis, as it is a safer and gentler method compared with chemical hydrolysis with strong acid or alkali. Industrial applications using acid or alkaline hydrolysis are cost- and time-effective, but the harsh chemicals can reduce a food product’s nutritional qualities and produce ecologically unacceptable pollution [16,17]. Additionally, He et al. [18] suggested that the natural antioxidants produced when protein is enzymatically hydrolyzed can reduce the risk of chronic diseases, including cancer and cardiovascular disease. There are artificial antioxidants added commercially for preventing lipid oxidation and extending the shelf-life of food products [19]; however, most consumers have negative feelings about artificial antioxidants due to questions about their safety [20,21]. Protein hydrolysates are natural products with antioxidant properties that may be beneficial to health by reducing the oxidation of food. The antioxidant activity of protein hydrolysates depends on several factors, including the DH, the presence of free amino acids (FAAs), nitrogen solubility, and the type of enzyme used in the hydrolysis process. The applications of soybean protein hydrolysates with antioxidant properties are more common in the food industry to improve the quality of functional food, add nutritional value, and facilitate food processing, utilization, and marketing [18]. Therefore, in this study, we investigated the functional and antioxidant properties of an enzymatic preparation of soy protein hydrolysates.

## 2. Results and Discussion

### 2.1. Optimization of Hydrolysis Conditions

**Figure 1:**
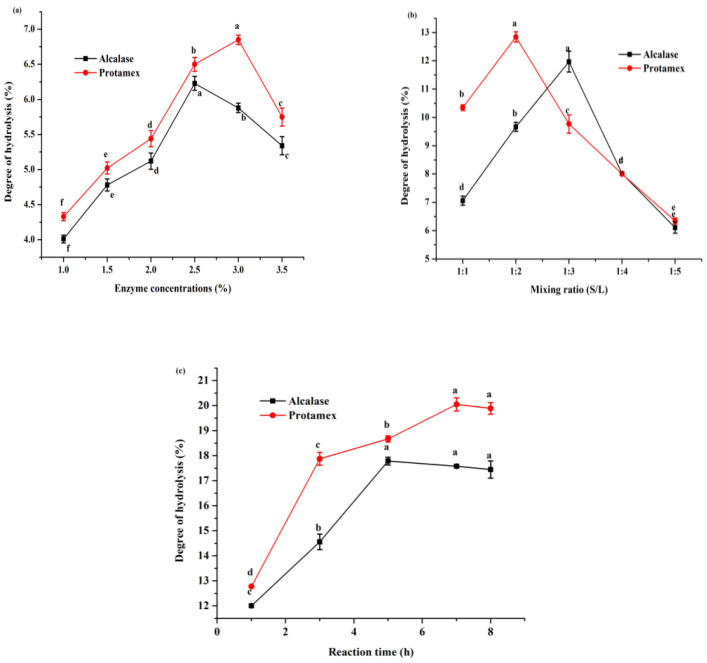
Effects of different conditions on degree of hydrolysis: (**a**) enzyme-to-substrate ratio, (**b**) sample-to-buffer ratio (S/L), and (**c**) time of incubation. Data are expressed as mean ± S.D. for *n* = 3. Different small letters within each assay indicate significant differences (*p* < 0.05).

The two tested proteases produced peptides with different DHs under various conditions and showed a significant correlation with enzyme concentration, the sample mixing ratio, and time (Figure 1a–c). The effect of the E/S ratio on the DH was determined at 1, 1.5, 2, 2.5, 3, and 3.5% (based on the protein content in the substrate) (Figure 1a). At an Alcalase and Protamex enzyme concentration of 1% (*w*/*w*), the DH was 4.01% and 4.33%, respectively. Increasing the concentration of the enzymes to 2.5% and 3% increased the E/S ratio above the optimal levels but did not result in a significant change in the DH. Similarly, the effect of the soy protein/buffer ratio on the DH was examined at 1:1, 1:2, 1:3, 1:4, and 1:5 (Figure 1b). Furthermore, the effect of incubation time was analyzed at 1, 3, 5, 7, and 8 h (Figure 1c). The DH was significantly increased with increased sample mixing and time under optimum conditions. However, after increasing the sample/buffer ratio, enzyme concentration, and time over the optimal ratio for Alcalase (1:3, 2.5%, 5 h) and Protamex (1:2, 3%, 7 h), the DH values were not significantly different. This result could be due to enzyme aggregation or caused by the inhibition of substrate diffusion, with the resulting saturation of the reaction rate [22]. The hydrolysates generated by Protamex achieved the highest DH value (20.05%) at 7 h of incubation (Figure 1c), followed by Alcalase (17.79%) at 4.5 h incubation (Figure 1c), with a significance level of *p* < 0.05. Therefore, the optimal conditions for further experiments were chosen as follows: E/S 2.5% for Alcalase and 3% for Protamex; the sample mixing ratio, 1:3 for Alcalase and 1:2 for Protamex; and reaction time, 5 h for Alcalase and 7 h for Protamex.

### 2.2. Average Yield of Soybean Protein Hydrolysate

The average hydrolysate yields from the freeze-dried soybean protein under optimal conditions are presented in Table 1. The yield was strongly related to the DH, with the highest yield (19.77%) observed for Protamex, followed by 16.08% for Alcalase. The difference between these yields could be due to the differences in enzyme activity and temperature. Our yield of the hydrolyzed soy protein was lower than that of Jamdar, Rajalakshmi, Pednekar, Juan, Yardi, and Sharma [23], who analyzed the formation of the peanut protein hydrolysate by using Alcalase.

### 2.3. Proximate Composition 

The proximate composition of the non-hydrolyzed (soybean flour, without enzyme treatment) and hydrolyzed protein is displayed in Table 1. The hydrolysate contained a higher protein concentration (51.64%) than the non-hydrolyzed material (48.09%). The non-hydrolyzed protein extract had the highest fat content (17.69%), followed by the Protamex-hydrolyzed sample (11.03%), with a significant difference. In contrast, the Protamex digest was revealed to contain significantly lower levels of ash (4.47%) than the Alcalase and non-hydrolyzed samples. With regard to moisture, the non-hydrolyzed protein samples had a relatively high content (8.95%), compared with the Alcalase and Protamex hydrolysates, which was in agreement with prior results [23]. This could be because the dissolution of protein during hydrolysis could have significantly reduced the fat content, which would be lost during the sample’s centrifugation to separate insoluble and undigested substances [24].

### 2.4. Molecular Weight (Mw) Profiles

The molecular weight distribution of the non-hydrolyzed samples and the Alcalase and Protamex hydrolysates obtained from soybean under optimal conditions are displayed in Table 2. The protein hydrolysates were mainly composed of small Mw fractions (<1000 Da), with percent distributions of 94.86% for Protamex and 92.94% for Alcalase, while the non-hydrolyzed protein had a content of only 0.41% (<1000 Da). Islam et al. [24] reported that the dietary proteins rich in small Mw peptides (˂1000 Da) could be more available in the food system and contribute more to the nutritional value. Additionally, it has been shown that low-molecular-mass peptides enhance antioxidant activity [25,26,27]. 

### 2.5. Amino Acid Profiles

In the current study, the total amino acids evaluated in the hydrolysate and non-hydrolyzed (dried powder) samples of soybean are shown in Table 3. The major amino acids in the Protamex digest were aspartic acid (5.78%), glutamic acid (8.91%), leucine (3.55%), and lysine (4.13%). Aspartic acid and glutamic acid are the most important amino acids that contribute to palatability. In addition, alanine, glycine, serine, and threonine taste sweet, while arginine, leucine, isoleucine, methionine, phenylalanine, histidine, and valine have a bitter taste [28]. The non-hydrolyzed soy protein was richer in histidine (1.32%), isoleucine (2.01%), and valine (1.59%) than the hydrolyzed protein. This result may be because the proteins were more prone to not breaking down into peptides with Mw distribution during the enzymatic hydrolysis process. However, the essential amino acids were higher than the suggested requirements by the FAO/WHO [29] for adults, except for the content of methionine+ cysteine (non-hydrolyzed), while the evaluated essential amino acids were slightly lower for a child (Table 3).

### 2.6. Functional Properties of Soybean Protein Hydrolysate

#### 2.6.1. Protein Solubility

**Figure 2:**
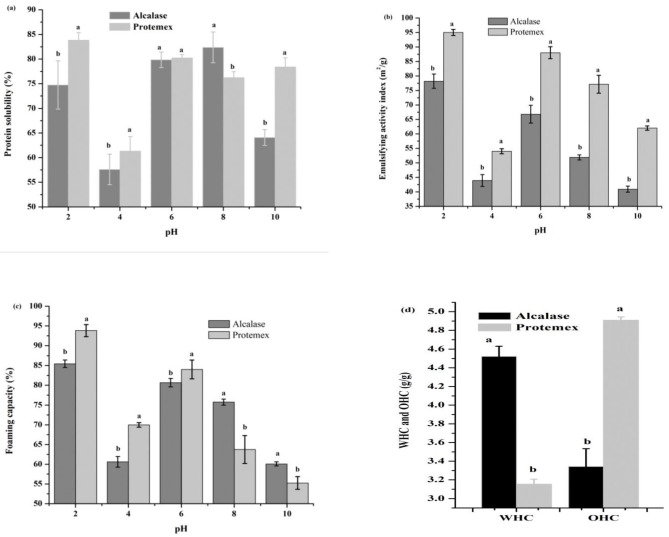
Functional properties of soybean protein hydrolysate: (**a**) protein solubility (%), (**b**) emulsifying activity index (m^2^/g), (**c**) foaming capacity (%), and (**d**) water- and oil-holding capacity (g/g). The values represent means of three independent experiments ± SD. Different characters indicates significant differences at each pH (*p* < 0.05).

The solubility of ingredients is an important functional property that is required in the food industry for many different applications in product manufacturing [30]. The solubility rates of the soybean protein hydrolysate with different DHs at pH levels of 2, 4, 6, 8, and 10 are shown in Figure 2a. The highest protein solubility (83.83%) was obtained for Protamex at pH 2. These results are attributable to the DH and small molecular weights (<1000 Da) of this sample and are supported by the results of Naqash and Nazeer [31] who showed that digestion of proteins to smaller peptides led to greater solubility. The lowest value (57.61%) was obtained at pH 4 for the Alcalase hydrolysate. Foh et al. [32] reported that pH 4.5 and 5.5 occur near the isoelectric point (pI) at which the net charge of the original proteins is minimized, and consequently, more protein–protein interactions and fewer protein–water interactions occur. The authors suggested that soybean protein hydrolysates had high solubility, which could result in improved appearance and smooth mouth feel of the food and food products [30]. 

#### 2.6.2. Emulsification Properties

The emulsification activity index (EAI) of the soybean protein hydrolysate with various DHs (20.05% and 17.79%) is presented in Figure 2b. The results showed that the EAI was significantly affected by pH, as the EAI was the highest for Protamex at pH 2 (95.03 m^2^/g) and was the lowest at pH 4 (54.02 m^2^/g). The Alcalase hydrolysate had the lowest EAI at pH 10 (40.93 m^2^/g), while the highest value was 78.20 m^2^/g at pH 2. These results may be due to protein solubility and the small size of the peptides [33] reported that the emulsifying properties of enzymatically digested proteins were predominantly influenced by protein solubility, molecular size, and DH. The lowest EAI of soybean protein hydrolysate was at pH 4, possibly due to more protein interactions [31]. 

#### 2.6.3. Foaming Capacity 

**Figure 3:**
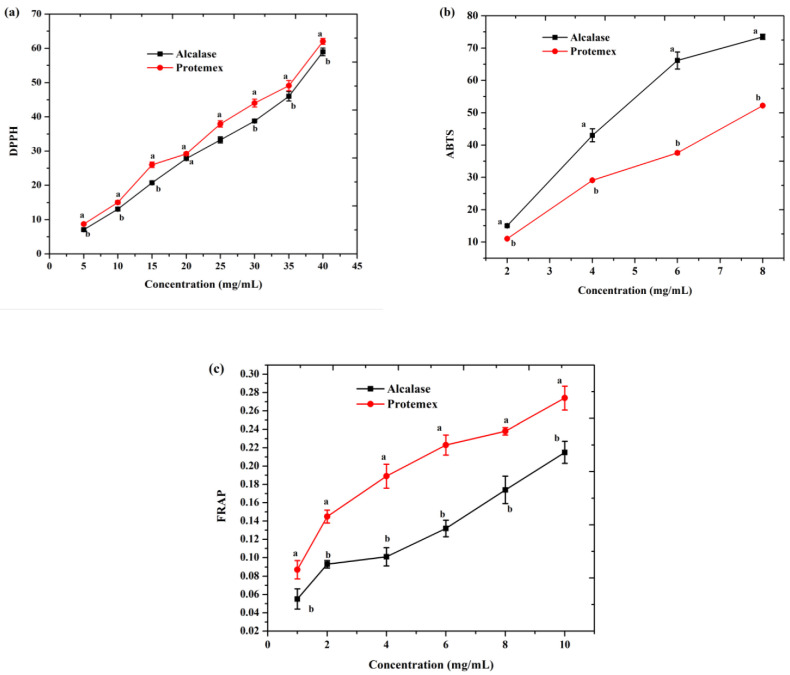
Comparison of antioxidant activities of soybean protein hydrolysates at different DH: (**a**) DPPH free radical scavenging activity (%), (**b**) ABTS free radical scavenging activity (%), and (**c**) reducing power capacity at 700 nm. Values are mean ± SD. Different small characters indicate significant differences (*p* < 0.05).

The foaming capacity of the soybean protein hydrolysates was evaluated by different enzymatic treatments (Figure 2c). The highest foaming capacity for the Protamex hydrolysate was 93.84% at pH 2.0, closely followed by Alcalase at the same pH (85.45%) (Figure 3c). The highest foaming stability was at pH 2 and the lowest at pH 4, which may be due to the low protein solubility near the isoelectric points at pH 4 [31]. Chalamaiah et al. [33] mentioned that the foaming capacity and stability increased with the higher protein content from using the Alcalase enzyme. In addition, high foaming properties mainly depend on the transportation, permeation, and redisposition of the molecules at the water/air interface.

#### 2.6.4. Water- and Oil-Holding Capacity

The water-holding capacity (WHC) and oil-holding capacity (OHC) are affected by peptides and amino acids. The WHC and OHC of soybean protein hydrolysates were evaluated, and the highest value of the WHC (4.52 g water/g protein hydrolysate) was achieved for Alcalase, followed by Protamex in the 3.16 g water/g protein hydrolysate (Figure 2d). The Protamex hydrolysate achieved the highest OHC in the 4.91 g oil/g protein hydrolysate, followed by Alcalase in the 3.34 g oil/g protein hydrolysate. The WHC is affected by a high DH, which results in smaller-molecular-weight peptides, thus decreasing the WHC [34]. Santos, Martins, Salas-Mellado, and Prentice [34] found that the OHC ranged from 3.86 to 5.12 mL oil/g protein for the blue-winged sea robin hydrolysates obtained at a DH of 15% with Alcalase enzyme. The authors suggested that the OHC is an important functional property that affects the flavor of food products. 

### 2.7. Antioxidant Properties

#### 2.7.1. DPPH Free Radical Scavenging Capacity

The DPPH free radical scavenging activities of the soybean protein hydrolysates from the two enzymatic treatments are presented in Figure 3a. Protamex achieved the highest DPPH activity at 62.07%, followed by Alcalase (59.07%) at a protein concentration of 40 mg/mL. These results may be connected with DH, peptide size, and the presence of amino acids that are electron donors and may react with free radicals to convert them to more stable products. Park et al. [35] reported that amino acids such as isoleucine, threonine, valine, and other hydrophobic amino acids strongly contribute to a positive DPPH scavenging capability.

The IC_50_ value of Protamex was 33.58 ± 1.03 mg/mL, which was stronger than that of the Alcalase hydrolysates at 38.28 ± 0.92 mg/mL. However, the DPPH antioxidant activity of the soybean protein hydrolysates was significantly lower than that of the standard BHT (IC_50_ = 4.2 ± 0.06 mg/mL). The DPPH radical scavenging activity was lower than that of the housefly larvae hydrolysate using Alcalase 2.4 L and Flavourzyme [36]. 

#### 2.7.2. ABTS Free Radical Scavenging Activity

The ABTS radical assay is an excellent tool for the evaluation of antioxidant activity, in which the radical reacts to form an ABTS radical complex [37]. As clearly shown in Figure 3b, the Alcalase hydrolysates had the highest ABTS radical scavenging activity (70.21%), followed by Protamex (52.31%) at a protein concentration of 8 mg/mL, with a significant difference (*p* ≥ 0.05). Hassan, Xavier, Gupta, Nayak, and Balange [38] reported that some amino acids (cysteine, tryptophan, tyrosine, and histidine) showed better ABTS scavenging activity. It was also found that the small-molecular-weight peptides were mainly responsible for the antioxidant activity [27]. These findings were closely related to the current research results (Table 2 and Table 3). 

The IC_50_ values for the ABTS radicals with Alcalase and Protamex hydrolysates were 5.02 ± 0.08 and 7.50 ± 0.11 mg/mL, respectively, which were lower in antioxidant activity than that of BHT (IC_50_ = 0.21 ± 0.01 mg/mL). 

#### 2.7.3. Reducing Power 

The ferric ion-reducing antioxidant power (FRAP) of the soybean protein hydrolysates is displayed in Figure 3c. The results showed that the FRAP significantly increased (*p* < 0.05) as the protein hydrolysate concentration increased. The highest Protamex hydrolysate absorbance value was 0.27 unit, followed by Alcalase (0.22 unit) at a protein concentration of 10 mg/mL. The higher the absorbance value, the higher the reducing power of the protein hydrolysate. The strong reducing power capability of Protamex may be attributed to the presence of protons and electrons generated during peptide cleavages. The soybean protein hydrolysates were lower in reducing power than the buckwheat protein hydrolysate [39]. In this study, the results were lower than those of the commercial BHT (0.31 unit at 0.2 mg/mL).

## 3. Materials and Methods

### 3.1. Sample Collection and Preparation 

Seeds of the BARI-5 variety of soybean (*Glycine max* L.) were obtained from the Bangladesh Agricultural Research Institute (BARI), Gazipur, Bangladesh. The seeds were washed thoroughly under running tap water, to remove husks and then milled by a miller (PMill 2000, Dhaka, Bangladesh). The soy powder was stored at −20 °C for further analysis.

### 3.2. Enzymes and Reagents

Alcalase 2.4 L (2.4 AU-A/g) from *Bacillus licheniformis* and Protamex (400 U/g) from Bacillus protease complex were procured from Nanjing Chengna Chemical Co., Ltd. (Nanjing, China). The enzyme was stored at 4 °C. DDPH (1,1-diphenyl-2-picrylhydrazyl), ABTS (2,2′-azino-bis(3-ethylbenzothiazoline-6-sulfonic acid)), SDS (sodium dodecyl sulfate), BHT, and potassium ferricyanide were purchased from Shanghai Yuanye Biotechnology Co., Ltd. (Shanghai, China). All the other chemicals and reagents used in the experiments were of high purity and analytical grade. 

### 3.3. Preparation of Protein Hydrolysates

The protein hydrolysates were prepared following a previously published procedure by Islametal. [24], with some modifications. Soy protein was hydrolyzed using two kinds of protease, Alcalase, and Protamex, under optimal conditions, as shown in Table 4. The buffers used were 25 mM sodium phosphate (pH 6.0 to 7.0), and Tris-HCl (pH 7.5 to 9.0). The enzyme activity was stopped by heating the mixture at 90 °C for 20 min in a water bath (WNB-14, Buechenbach, Germany), after which the mixture was immediately transferred to an ice bath to cool and centrifuged (ST 40R, Thermo Electron LED GmbH, Langenselbold, Germany) at 10,000× *g* for 10 min at 4 °C. The supernatants were collected and freeze-dried under vacuum at −48 °C (Scientz-10N, Ningbo Scientz Biotech. Co., Ltd., Zhejiang, China), and protein hydrolysates were stored at −20 °C for later analysis.

### 3.4. Determination of Degree of Hydrolysis (DH)

The degree of hydrolysis (DH) was measured using a formal titration method as described by Islam et al. [22], with minor modifications. Briefly, 1.5 g of lyophilized protein hydrolysate was dissolved in deionized water, and the volume was made up to 50 mL. The solution was adjusted to pH 7.0 with 0.1 N NaOH, 10 mL of 38% (*v*/*v*) formaldehyde was added, and the solution was kept for 5 min at room temperature (25 °C). Titration was conducted to the end point at pH 8.5 using standard 0.1 N NaOH, and the volume consumed was used to calculate the number of free amino groups (FAGs). The total nitrogen (TN) in the sample was determined by using the Kjeldahl method following the standard procedure [40]. The FAGs and DH were calculated according to the following equations:

$$\mathrm{FAGs}\;\left(\%\right)=\left[\frac{V\times C\times \frac{14.007}{1000}}{S}\right]\times 100$$

$$\mathrm{DH}\;\left(\%\right)=\left[\frac{\%\mathrm{FAG}}{\%\mathrm{TN}}\right]\times 100$$

where V = mL of 0.1 N NaOH used; C = the concentration of sodium hydroxide used for titration (0.1 N); S = amount of sample (g); and TN = total nitrogen in the sample.

### 3.5. Determination of Yield 

The yield of soybean protein hydrolysate was determined according to a modified procedure [23] and calculated using the following equation:

$$\;\mathrm{Yield}\;\left(\%\right)=\frac{\mathrm{Weight}\;\mathrm{of}\;\mathrm{protein}\;\mathrm{hydrolysate}\;\mathrm{powder}\;\left(g\right)}{\mathrm{Weight}\;\mathrm{of}\;\mathrm{raw}\;\mathrm{sample}\left(g\right)}\times 100$$

### 3.6. Determination of Proximate Composition 

Proximate compositions such as the moisture, protein, fat, and ash content of the non-hydrolyzed dried soybean powder and soybean protein hydrolysates were measured using the standard guidelines of the Association of Official Analytical Chemists (AOAC) [40]. The moisture content was determined by drying the samples in an oven at 105 °C until a constant weight was obtained. The total nitrogen content was determined by using the standard micro-Kjeldahl method, and the amount of crude protein was calculated by multiplying the total nitrogen (N%) by the factor of 6.25. The ash content was evaluated through the incineration of the samples at 600 °C in a muffle furnace until only white ash remained. The fat content was measured using a macro-Soxhlet apparatus (SZG-101, Shanghai, China) with petroleum ether. 

### 3.7. Determination of Molecular Weight (Mw) Distribution 

The molecular weight (Mw) distributions of the dried non-hydrolyzed sample (soybean flour) and protein hydrolysates were determined according to the method of Tong et al. [41], with slight modifications. Briefly, 100 mg of non-hydrolyzed soybean or protein hydrolysate was transferred into 15 mL glass tubes, and 10 mL of ultrapure water was added. The glass tubes were placed in an ultrasonic bath for 5 min and then quickly transferred into centrifuge tubes and centrifuged at 10,000× *g* for 10 min (SClogec D3024R, Beijing, China) at 4 °C. The supernatants were filtered and used for molecular weight profile determination via gel permeation chromatography using an HPLC system (Waters-1525, Milford, MA, USA). The TSK-Gel 2000 SWXL (300 × 7.8 mm) column (Tosoh, Japan, Tokyo) was equilibrated with the mobile phase composed of acetonitrile/water/trifluoroacetic acid 45/55/0.1 (*v*/*v*). The column was eluted at a temperature of 30 °C and a flow rate of 0.5 mL per min and monitored at 220 nm. Cytochrome C (12,384 Da), bacitracin (1422 Da), Gly-Gly-Try-Arg (GGYR) (451 Da), and Gly-Gly-Gly (GGG) (189 Da) were used as the molecular weight standards.

### 3.8. Analysis of Amino Acid Profiles

Amino acid analysis was performed according to the method of Islam, Hongxin, Admassu, Mahdi, Chaoyang, and Wei [22], with minor modifications. The protein hydrolysate (100 mg) and non-hydrolyzed protein (100 mg) were dissolved in 8 mL of 6 M HCl under nitrogen gas and heated in an oven at 120 °C for 22 h. The samples were neutralized by adding 4.8 mL of 10 M sodium hydroxide and centrifuged at 10,000× *g* for 10 min at 4 °C. Aliquots of 1 μL of centrifuged supernatants were injected into an HPLC analytical column of 250 × 4.6 mm I.D, 5 μm particle size (Agilent Technologies, Palo Alto, CA, USA). The analysis was run on an RP-HPLC (HP-Agilent 1100 model, Agilent Technologies) system at a column temperature of 40 °C and a flow rate of 1.0 mL/min, with detection at 338 nm. Mobile phase A was 7.35 mM sodium acetate/tri-ethylamine/tetrahydrofuran (500:0.12:2.5, *v*/*v*/*v*) adjusted to pH 7.20 ± 0.05 with acetic acid, and mobile phase B (pH 7.20 ± 0.05) was 7.35 mM C_2_H_3_NaO_2_/CH_3_OH/acetonitrile (1:2:2, *v*/*v*/*v*). 

### 3.9. Functional Properties of Soybean Protein Hydrolysate

#### 3.9.1. Protein Solubility

The solubility of the protein hydrolysate was evaluated according to the procedure of Jamdar, Rajalakshmi, Pednekar, Juan, Yardi, and Sharma [23], with slight modifications. Briefly, 200 mg of the hydrolysate was dissolved in 20 mL of ultrapure water, and the solution was adjusted to pH 2, 4, 6, 8, and 10 with 0.1 M HCl or 0.1 M NaOH. The solutions were incubated at 30 °C with stirring (Blue Pard, Yiheng Technical Co., Ltd., Shanghai, China) at 150 rpm for 25 min and then centrifuged at 10,000× *g* for 10 min at 4 °C, and the supernatants were recovered (SCLOGEX- D3024R, Beijing, China). The protein content of the supernatant was measured by using the Kjeldahl method [42], and the protein solubility was calculated as:

$$\mathrm{Protein}\;\mathrm{solubility}\;\left(\%\right)=\frac{\mathrm{Protein}\;\mathrm{content}\;\mathrm{in}\;\mathrm{supernatant}}{\mathrm{Total}\;\mathrm{protein}\;\mathrm{content}\;\mathrm{in}\;\mathrm{sample}\;}\times 100$$

#### 3.9.2. Emulsifying Properties

The emulsifying activity index (EAI) of soybean protein hydrolysates was determined by using the method of [43], with minor modifications. Solutions of 1% soybean protein hydrolysates were adjusted to pH 2, 4, 6, 8, and 10 and then mixed with 10 mL of soybean oil and homogenized at 18,000 rpm at 4 °C for 1 min. At 0 and 10 min after homogenization, 50 μL samples were pipetted from the bottom and diluted with 5 mL of 0.1% sodium dodecyl sulfate (SDS) (*w*/*v*). The absorbance of the solutions was measured at 500 nm with a spectrophotometer (Shanghai, China). The EAI was calculated by the following formula:

$$E\mathrm{AI}\;\left(m^{2}g^{-1}\right)=\frac{2\times 2.303\times A_{500}}{\emptyset \times W}$$

where A_500_ = absorbance at 500 nm, W = weight of sample (g), and ∅ = oil volume fraction (0.25). 

#### 3.9.3. Water- and Oil-Holding Capacity, Foaming Capacity

The water-holding capacity (WHC) and oil-holding capacity (OHC) were evaluated using the procedure of Noman et al. [44], with slight modifications. Briefly, each protein hydrolysate (0.5 g) was dissolved in 10 mL of deionized water and 10 mL of soybean oil in a centrifuge tube and dispersed with a vortex mixer (XW-80A, Hangzhou China) for 1 min. The water dispersion was allowed to stand for 6 h, while the oil dispersion stood for 25 min at 25 °C, and then both were centrifuged at 5000× *g* for 25 min at 4 °C. The water-holding capacity was obtained by filtering the supernatant through Whatman No. 1 filter paper and calculating the difference in weight. The amount of free oil remaining was measured to obtain the oil-holding capacity from the difference in weight.

The foaming capacity (FC) was determined according to the method of Jamdar, Rajalakshmi, Pednekar, Juan, Yardi, and Sharma [41], with some modifications. The soybean protein hydrolysate (1 g) was dissolved in 100 mL of deionized water and adjusted to pH 2, 4, 6, 8, and 10 with 0.1 N HCl or NaOH. The adjusted solutions were poured into a 250 mL volumetric cylinder, and foam was produced by using a homogenizer (Morgec MBL50, Shanghai, China) at 16,000 rpm for 2 min. The volume of the foam was recorded immediately after homogenization. The FC was calculated according to the following equation:

$$\mathrm{FC}=\frac{V_{a}-V_{b}}{V_{b}}\times 100$$

where V_a_ is the volume in mL before homogenization, and V_b_ is the volume afterward. 

### 3.10. Antioxidant Properties

#### 3.10.1. DPPH Radical Scavenging Activity

The 2,2-diphenyl-1-picrylhydrazyl (DPPH) radical scavenging activity was evaluated by using the protocol of Jamdar, Rajalakshmi, Pednekar, Juan, Yardi, and Sharma [23]. Briefly, 2 mL of the protein hydrolysate at concentrations of 5, 10, 15, 20, 25, 30, 35, and 40 mg/mL was added to 2 mL of 0.16 mM DPPH in methanol. The mixture was vortexed for 1 min and left to stand at room temperature for 30 min in the dark. BHT was used as a positive control, and the absorbance was read at 517 nm. The DPPH scavenging activity was calculated using the following equation:

$$\mathrm{DPPH}\;\mathrm{scavenging}\;\mathrm{activity}\;(\%)=\frac{{\mathrm{Ab}}_{1}-{\mathrm{Ab}}_{2}}{{\mathrm{Ab}}_{1}}\times 100$$

where Ab_1_ is the absorbance without the sample, and Ab_2_ is the absorbance with the sample. 

#### 3.10.2. ABTS Radical Scavenging Activity

The ABTS free radical scavenging activity was determined by using the method of Liu et al. [45], with some modifications. Briefly, the BTS free radical solution was generated by mixing 2.45 mM ABTS with 7 mM potassium persulfate and keeping the solution in the dark at room temperature for 16 h. The ABTS radical stock solution was diluted in 5 mM phosphate-buffered saline at pH 7.4 to an absorbance of 0.75 ± 0.02 at 734 nm. The diluted ABTS^•+^ solution was mixed with 100 µL of the different concentrations of the soybean protein hydrolysate (2, 4, 6, and 8 mg/mL) and incubated at room temperature for 10 min in the dark. The absorbance of the solution at 734 nm was measured, with BHT used as a positive control. The ABTS scavenging activity was calculated by using the following equation:

$$\mathrm{ABTS}\;\mathrm{scavenging}\;\mathrm{activity}\;(\%)=\frac{{\mathrm{Ab}}_{1}-{\mathrm{Ab}}_{2}}{{\mathrm{Ab}}_{1}}\times 100$$

where Ab_1_ is the absorbance without the sample, and Ab_2_ is the absorbance with the sample. 

The IC_50_ values of the protein hydrolysates for scavenging DPPH and ABTS were determined from the linear regression analysis of the plot of concentration vs. percent inhibition. 

#### 3.10.3. Ferric Reducing Antioxidant Power (FRAP)

The FRAP was determined according to the procedure described by Hassan et al. [38], with minor modifications. Briefly, 2 mL of the soybean protein hydrolysate at different concentrations (1, 2, 4, 6, 8, and 10 mg/mL) was mixed with 2 mL of a phosphate buffer (200 mM, pH 6.6), and 2 mL of 1% potassium ferricyanide was added. The mixture was mixed vigorously by vortexing (XW-80A, Hangzhou, China) for 1 min and incubated at 50 °C for 25 min. Then, 1 mL of 10% TCA was added, mixed, and centrifuged at 10,000× *g* for 10 min at 4 °C. The supernatants were collected and mixed with 2 mL of ultrapure water and 0.4 mL of 0.1% FeCl_3_. Lastly, the mixture was held for 10 min at room temperature, and the absorbance at 700 nm was measured with a spectrophotometer. BHT was used as a positive control. 

### 3.11. Statistical Analysis

All the experiments were performed at least in triplicate (*n* = 3). The results were subjected to a one-way analysis of variance (ANOVA). Duncan’s new multiple-range test was used to determine whether the differences between the samples were statistically significant within the 95% confidence interval (*p* < 0.05) using the IBM SPSS 22.0 software (SPSS Inc., Chicago, IL, USA).

## 4. Conclusions

The degree of hydrolysis and the functional and antioxidant properties of the soybean protein hydrolysates were significantly affected by enzymatic hydrolysis conditions. Protein solubility was found to be related to peptide molecular weight and amino acid composition. The highest value of the EAI was achieved at pH 2.0, and the lowest value was at pH 4.0, while the foaming capacity was the highest at pH 2.0 for the Protamex hydrolysate. The pH level and DH significantly affected the foaming properties. The best WHC was achieved by the Alcalase hydrolysate, while the Protamex hydrolysate had the highest OHC. The soybean protein hydrolysates showed high antioxidant activities against DPPH and FRA for Protamex, while the ABTS radical scavenging activity was more pronounced with the Alcalase hydrolysate. Therefore, soybean protein hydrolysates have good functional and antioxidant properties, which could be applicable in the food and pharmaceutical industries. Further studies on the purification and identification of specific peptides and amino acid sequences are needed for improving the quality of functional foods.

## Figures and Tables

**Table 1:** Proximate composition of non-hydrolyzed and hydrolyzed soybean protein (%) and yield (%) (dry weight).

Parameters	Non-Hydrolyzed	Alcalase	Protamex
Protein	48.09 ± 2.13 ^b^	50.21 ± 1.32 ^a^	51.64 ± 1.23 ^a^
Moisture	8.95 ± 0.34 ^a^	7.66 ± 0.18 ^b^	6.99 ± 0.43 ^bc^
Fat	17.69 ± 0.48 ^a^	10.07 ± 0.68 ^c^	11.03 ± 0.08 ^b^
Ash	5.03 ± 0.22 ^a^	4.78 ± 0.29 ^b^	4.47 ± 0.65 ^b^
Yield	-	16.08 ± 0.42 ^b^	19.77 ± 0.88 ^a^

The values represent means of three independent experiment ± SD. Different letters (a,b,c) indicates significant differences at the each pH level (*p* < 0.05).

**Table 2:** Molecular mass distribution of non-hydrolyzed soybean protein and Alcalase, and Protamex hydrolysates. The spectra were obtained via HPLC (dry weight basis).

Mw (Da)		Content (%)	
	Non-Hydrolyzed	Alcalase	Protamex
17,000–15,000	93.07	0.21	0.43
7000–6000	3.97	0.38	0.67
4000–3000	1.04	0.53	0.42
2500–2000	0.87	1.15	0.88
1500–1000	0.64	4.79	2.74
700–600	0.25	19.21	9.75
300–200	0.13	42.82	34.98
<150	0.03	30.91	50.13

**Table 3:** Amino acid composition (g/100 g sample) of non-hydrolyzed soybean protein and protein hydrolysates (dry weight basis) in children vs. adults.

Parameters	Non-Hydrolyzed	Alcalase	Protamex	* FAO Requirements	
				Child	Adult
Essential amino acids					
Histidine	1.32 ± 0.03 ^a^	0.94 ± 0.01 ^b^	0.86 ± 0.02 ^c^	1.9	1.6
Methionine + cysteine	0.85 ± 0.01 ^b^	2.17 ± 0.06 ^a^	2.44 ± 0.09 ^a^	2.5	1.7
Phenylalanine + tyrosine	2.75 ± 0.05 ^a^	2.21 ± 0.11 ^b^	2.55 ± 0.13 ^b^	6.3	1.9
Threonine	1.44 ± 0.02 ^a^	1.36 ± 0.05 ^b^	1.59 ± 0.01 ^a^	1.4	0.9
Isoleucine	2.01 ± 0.07 ^a^	1.79 ± 0.04 ^c^	1.85 ± 0.05 ^b^	2.8	1.3
Leucine	3.48 ± 0.03 ^a^	3.42 ± 0.12 ^b^	3.55 ± 0.09 ^a^	6.6	1.9
Lysine	3.83 ± 0.05 ^b^	4.02 ± 0.17 ^a^	4.13 ± 0.10 ^a^	5.8	1.6
Valine	1.59 ± 0.01 ^a^	1.30 ± 0.03 ^b^	1.46 ± 0.07 ^a^	3.5	1.3
Non-essential amino acids					
Aspartic acid	5.15 ± 0.10 c	5.53 ± 0.23 ^b^	5.78 ± 0.21 ^a^	-	-
Glutamic acid	8.02 ± 0.15 ^c^	8.60 ± 0.31 ^b^	8.91 ± 0.34 ^a^	-	-
Serine	1.47 ± 0.03 ^b^	1.58 ± 0.04 ^b^	1.73 ± 0.07 ^a^	-	-
Glycine	2.09 ± 0.07 ^b^	2.15 ± 0.01 ^b^	2.36 ± 0.02 ^a^	-	-
Arginine	2.14 ± 0.02 ^a^	2.04 ± 0.06 ^b^	2.23 ± 0.17 ^a^	-	-
Alanine	2.60 ± 0.08 ^b^	2.88 ± 0.10 ^a^	3.03 ± 0.13 ^c^	-	-
Proline	1.13 ± 0.01 ^b^	1.09 ± 0.05 ^b^	1.45 ± 0.08 ^a^	-	-

* FAO/WHO (36). Data are expressed as mean ± SD for *n* = 3. Different letters show significant differences at *p* < 0.05. FAO = Food and Agriculture Organization.

**Table 4:** Hydrolysis conditions for preparation of protein hydrolysates from soybean.

Hydrolysis Conditions	Units	Symbol	Proteases	
			Alcalase	Protamex
Incubation temperature	°C	T	50	55
pH	pH	pH	8.0	7
Enzyme/Substrate ratio (*w*/*w*)	%	E/S	1, 1.5, 2, 2.5, 3, 3.5	1, 1.5, 2, 2.5, 3, 3.5
Soy protein/buffer ratio	g/mL	S/L	1:1, 1:2, 1:3,1:4, 1:5	1:1, 1:2, 1:3,1:4, 1:5
Incubation time	h	H	1, 3, 5, 7, 8	1, 3, 5, 7, 8
Inactivation temperature	°C	T	90	90
Inactivation time	min	-	20	20

## Data Availability

Data are contained within the article.
